# Comparisons of Prostate Cancer Inhibitors Abiraterone and TOK-001 Binding with CYP17A1 through Molecular Dynamics

**DOI:** 10.1016/j.csbj.2015.10.001

**Published:** 2015-11-04

**Authors:** Fei Xiao, Maohua Yang, Youjun Xu, Wanwipa Vongsangnak

**Affiliations:** a School of Biology and Basic Medical Sciences, Medical College of Soochow University, 199 Ren ai Road, China-Singapore Industrial Park, Suzhou, JiangSu 215123, China; b Department of Zoology, Faculty of Science, Kasetsart University, Bangkok 10900, Thailand

**Keywords:** Prostate cancer, Molecular dynamics, CYP17A1, Abiraterone, TOK-001

## Abstract

Cytochrome P450 17A1 (CYP17A1) is associated in the steroid hormone biosynthesis in human. As cell proliferation of prostate cancer in response to androgen steroid, an inhibition of CYP17A1 becomes an alternative approach to inhibit biosynthesis of androgen and support treatment of prostate cancer.

However, biology-driven inhibitor development of prostate cancer is poorly elucidated. The aims of this study are to address structural differences at atomic-level between CYP17A1 and inhibitors i.e., abiraterone and TOK-001, and further investigate the effect of point mutation of CYP17A1 on the active site stability and the local interactions that are hydrophobic interaction and hydrogen bonding throughout molecular dynamics (MD) simulation. After performing multiple comparisons among four different complexes across CYP17A1 and inhibitors, interestingly TOK-001 oriented toward the active pocket and formed larger volume with I-helix of CYP17A1 than abiraterone, whereas abiraterone showed tighter binding and more active site stability. Considering on the effect of hydrophobic interaction and hydrogen bonding between abiraterone and CYP17A1, the key residues of Phe114, Ile371, Val482, and Asn202 were identified. This contributes into tight binding interactions; however abiraterone is effectively weakened along with the global conformation mobility increased in A105L mutation. Surprisingly, overall conformation of the CYP17A1 remained stable when bound to TOK-001. This basic knowledge can guide future experiments on design of efficient inhibitors for CYP17A1, which provides theoretical basis of androgen-dependent disease therapy.

## 1 Introduction

Cytochrome P450 17A1 (CYP17A1, P450c17) is a crucial enzyme which belongs to the cytochrome P450, family 17, subfamily A. It is a dual-function monooxygenase which plays role in steroid hormone biosynthesis in humans [1]. Currently, CYP17A1 is a target of interest for the treatment of breast and prostate cancer cells that proliferate in response to androgen and estrogen steroids [2], [3]. So far, there are many studies focusing on mutation in CYP17A1 for structural and functional analysis [4], [5], [6], [7], [8], [9], [10], [11], [12]. Most of studies are often targeted at position of 105 from alanine to leucine in CYP17A1 active site (A105L mutation) which results in enzyme activities changes [13], [14], [15]. Consequently, this A105L mutation resulted in influencing the androgen level, thus supposed to be contributed into the androgen-dependent prostate cancer development. Several years ago, inhibitors of CYP17A1 were designed without structural information which was supposed to bind the cytochrome P450 haem iron [16]. Nonetheless, it has been hampered to reveal fundamental structural features for effective CYP17A1 inhibition. Recently, DeVore and Scott presented binding structures and modes of CYP17A1, in the presence of either abiraterone or TOK-001 [17]. This report showed better understanding of the CYP17A1 catalytic mechanism. In brief, abiraterone acetate (abiraterone), it is an active form of USFD-approved prodrug of this CYP17A1 inhibitor which improves overall survival in men with metastatic castration-resistant prostate cancer (CRPC), including patients for whom the disease has been progressed following chemotherapy, with compounds, such as docetaxel and the androgen receptor blocker enzalutamide [18], [19], [20]. Abiraterone binds to the CYP17A1 haem iron active site [17] with high affinity for prevention of androgen production. Using this full inhibitor, however, it induces the steroid imbalance of human, and thus frequently leads to hypertension, hypokalemia, and adrenocortical insufficiency, which are required to be monitored and be treated with additional drugs [21]. Furthermore, there is a report that the full inhibition of CYP17A1 by abiraterone possibly allows the androgen precursors flow through a “backdoor” androgen biosynthesis pathway [22]. This provides a probable route that can permit cancer progression.

Concerning on TOK-001 (also called as β-(hydroxy)-17-(1H-benzimidazole-1-yl) androsta-5, 16-diene, galeterone, or VN/124-1), it is identified as a selective development of drug candidate which modulates multiple targets in the androgen receptor (AR) signaling pathway [23]. According to many *in vivo* antitumor efficacy studies, TOK-001 was identified as the first example of an anti-hormonal agent which is an inhibitor of androgen synthesis [24], [25], [26], [27]. Because of its impressive anticancer properties, TOK-001 was selected as a clinical candidate, and it is currently undergoing phase III clinical trials for CRPC. Notably, TOK-001 is both a CYP17A1 inhibitor and AR antagonist [25] and the similarity of these binding modes is probably the reason for this dual mechanism of action.

However, the different binding mechanism of prostate cancer inhibitors of abiraterone and TOK-001 with CYP17A1 in terms of atomic-level structural characterization is still poorly elucidated. The aims of this study are therefore to address structural differences at atomic-level between CYP17A1 and inhibitors i.e., abiraterone and TOK-001, and further investigate the effects of point mutation of CYP17A1 on local interactions that are hydrophobic interaction and hydrogen bonding between these two inhibitors throughout molecular dynamics (MD) simulation.

To explore, we initially collected three available complex structures of CYP17A1 and inhibitors, i.e., two complexes for abiraterone with wild type (WT) and mutant (MT) CYP17A1 and one complex for TOK-001 with WT CYP17A1. We then constructed additional complex for TOK-001 with MT CYP17A1. MD simulations were performed on these four complexes to analyze the stability of an individual complex. Multiple comparisons of these complexes were afterwards analyzed on active site stability of the enzyme, local interactions, i.e., hydrophobic interaction and hydrogen bonding. This basic knowledge of inhibitor binding characteristics and key residues contributions leads to better understanding on cytochrome P450 superfamily enzymes, so that desirable changes in their enzymatic activities may be achieved. This can guide future computational and experimental work on efficient inhibitor design for CYP17A1 in theoretical basis of androgen-dependent disease therapy.

## 2 Materials and Methods

**Fig. 1:**
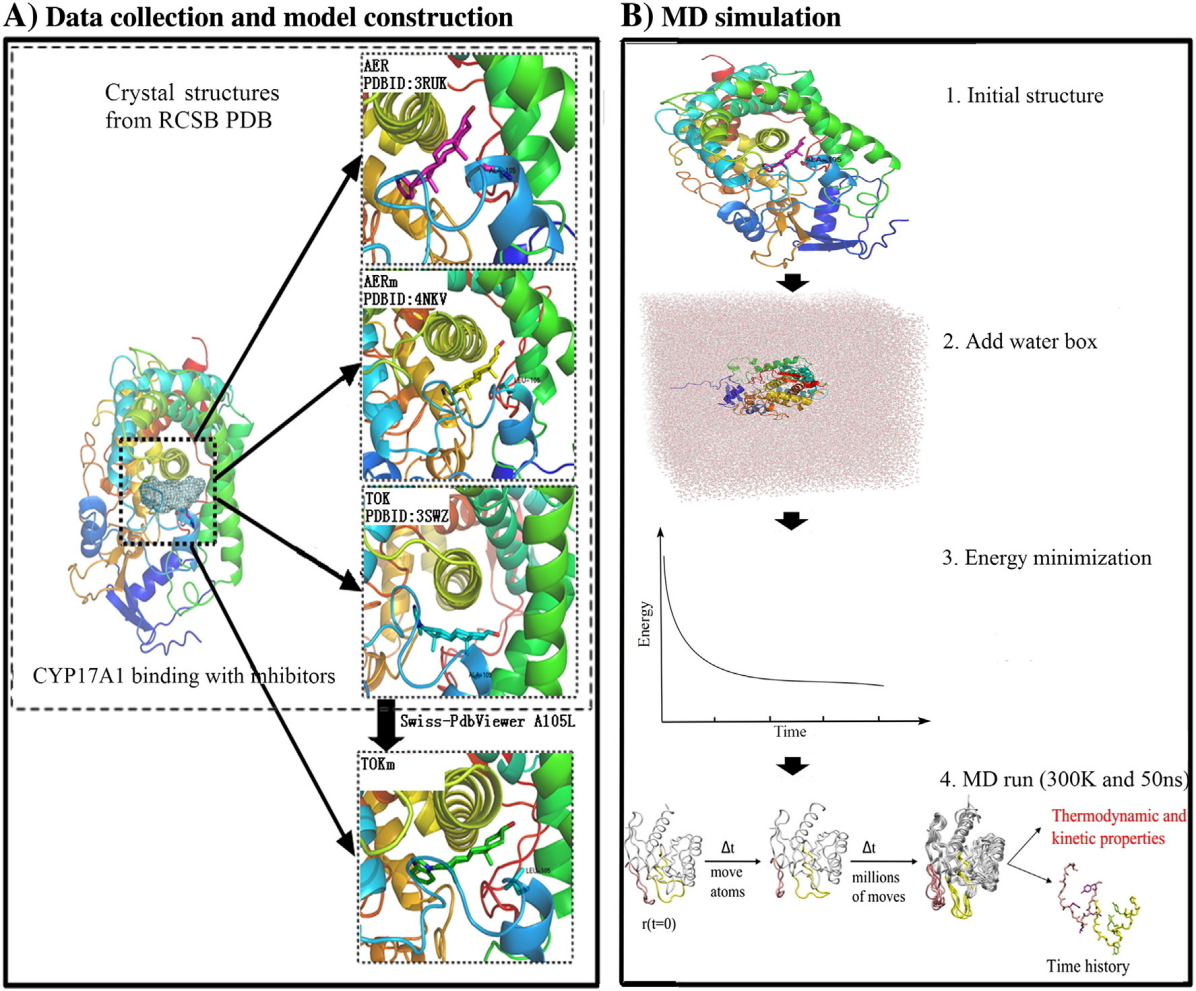
Overview of MD simulation method used for comparative analysis between prostate cancer inhibitors abiraterone and TOK-001 binding with CYP17A1. Illustration is divided into two steps, namely, data collection and model construction (A) and MD simulation (B).

An overview of the approach employed here for comparative analysis between prostate cancer inhibitors abiraterone and TOK-001 binding with CYP17A1 throughout MD simulation is depicted in Fig. 1. It is divided into two sections: 1) data collection and model construction, and 2) MD simulation. In this study, three complexes were retrieved from database that include a complex of abiraterone binding with WT CYP17A1 (AER), a complex of abiraterone binding with MT CYP17A1 (AERm), and a complex of TOK-001 binding with WT CYP17A1 (TOK). For additional complex of TOK-001 binding with MT CYP17A1 (TOKm), it was constructed throughout this study as described in the following.

### 2.1 Data Collection and Model Construction

As shown in Fig. 1A, the initial structures for AER, TOK, and AERm were retrieved from Research Collaboratory for Structural Bioinformatics, the Protein Data Bank (RCSB PDB) (www.rcsb.org/pdb/) (PDB ID code: 3RUK [17], 3SWZ [17], and 4NKV [15], respectively). It is noted that AERm has point mutation at position of 105 of CYP17A1 changing from alanine to leucine (A105L mutant). For TOKm, it was constructed through Swiss-PdbViewer [28] based on the X-ray structure of TOK and then CYP17A1 was mutated to obtain A105L mutant. The mutation of A105L was selected according to the key position in active site of CYP17A1 through the side chain interacting with inhibitors, which led to CYP17A1 functional deficiency. Concerning to all of these four complexes, only one monomer structure of enzyme and inhibitor were retained and prepared without haem. Hydrogen atoms were subsequently added to the initial structures with Chimera 1.10.1 [29]. The force field parameters of abiraterone and TOK-001 were supplied by GROMOS54A7 with Automated Topology Builder (ATB) and Repository 2.0 webserver [30].

### 2.2 MD Simulation

To obtain inhibitor and enzyme binding diversities at atomic level and the effects induced by a minor conformational change of the enzyme, MD simulation was carried out using GROMACS version 4.6.5 [31] with the standard of GROMOS96 force field parameter set 43a2 [32]. The procedure of our MD simulation is shown in Fig. 1B. First, initial structural complexes were solvated in a rectangular water box with single point charge water model [33] and were neutralized with chloride ions. To eliminate any unfavorable contacts, energy minimization was performed. Each complex was first minimized with the steepest descent algorithm by 5000 steps followed with L-BFGS algorithm [34], [35]. Subsequently, the minimized systems through MD run were simulated for 50 ns keeping temperature at 300 K and the pressure at 1 bar which maintained by Berendsen temperature and pressure coupling method [36]. LINCS algorithm [37] was obtained to constrain the hydrogen-contained bonds, and the Particle-Mesh Ewald (PME) [38] method was used to calculate the electrostatic interactions. To the end, the MD simulation trajectories were analyzed at the equilibrium state using the tools provided by GROMACS and the scripts written in this study. For illustration, PyMOL [39] and Chimera [29] were used.

## 3 Results and Discussion

**Fig. 2:**
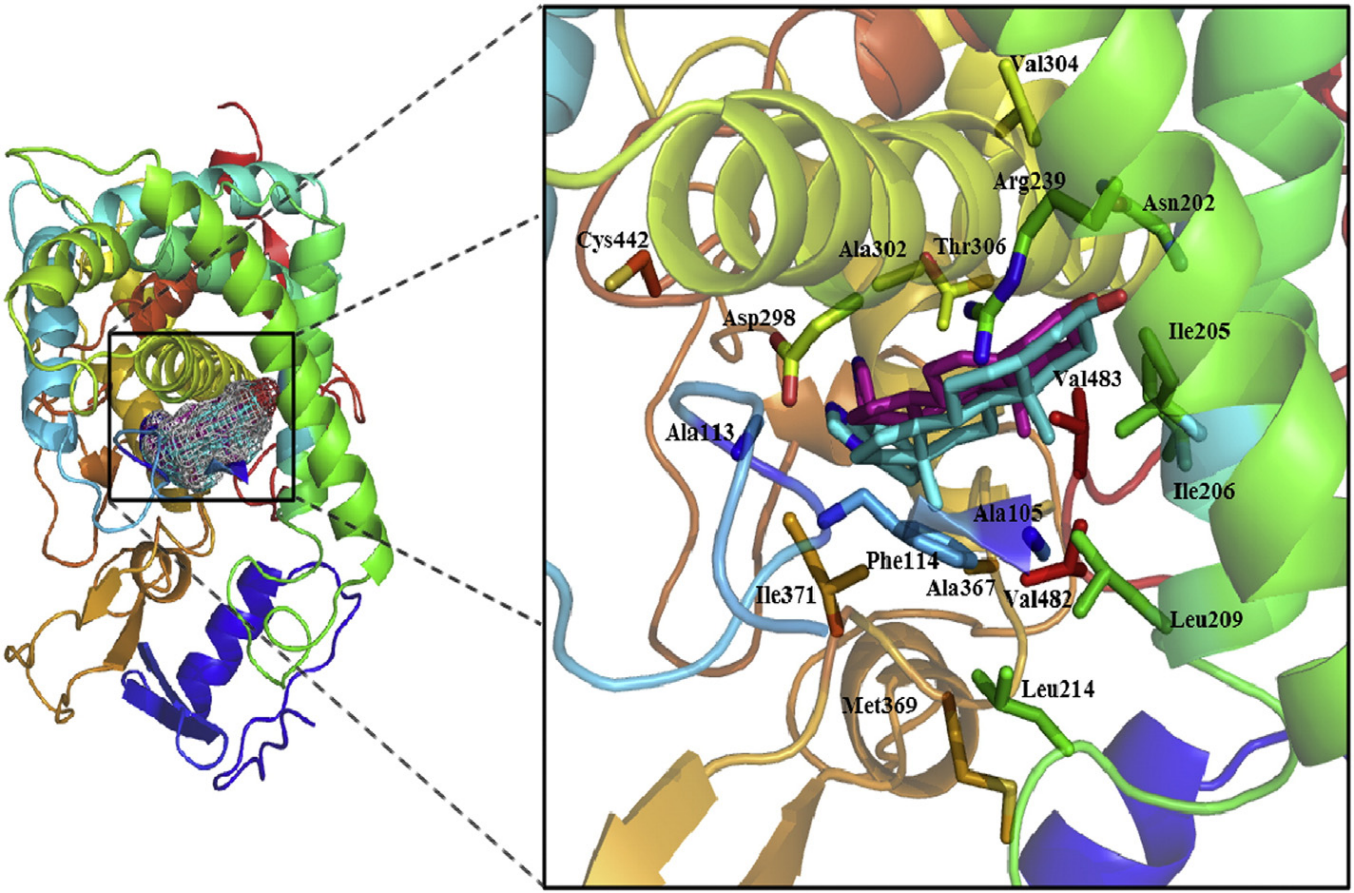
A colored representation of the CYP17A1-abiraterone/TOK-001 structure, Rainbow cartoon of CYP17A1 colored from the N-terminus (blue) to the C-terminus (red), abiraterone and TOK-001 are represented with purple and cyan sticks, respectively. Right box indicates the shared active site of abiraterone and TOK-001.

The overall structures and active sites of WT CYP17A1 bound to abiraterone (AER) and TOK-001 (TOK) were relatively conserved according to the X-ray structures, as shown in Fig. 2. These complex structures demonstrated the characteristics of cytochrome P450 fold and inhibitor binding mode. In details, the α-face packed against the I-Helix and formed a highly complementary hydrophobic planer surface with Gly301 and Ala302, while the β-face was primarily lined with hydrophobic atoms of Ala105, Ala113, Phe114, Ile206, Leu209, Val236, and Val482 (see Fig. 2). To gain insight into the binding mode and the influence of the position of 105 point mutation on the CYP17A1 binding with inhibitors, MT CYP17A1 bound to abiraterone (AERm) and TOK-001 (TOKm) were then studied and performed on MD simulation. So far, an application of MD simulation method has become indispensable in computational area with regards to enzyme and inhibitor interactions and enzyme conformation changes [40], [41]. In subsequent sections, the constructed model of TOKm is initially presented and the structural stability among four complexes (AER, AERm, TOK, and TOKm) and their multiple comparisons on active site stability, hydrophobic interaction, and hydrogen boding are later discussed.

### 3.1 Assessment of the Constructed Model of TOKm

To assess the constructed model of TOKm, the TOK-001 remained the unsubstituted α-face against the I-Helix, and the β-face toward the active pocket characterized by residues Leu105, Ala113, Phe114, Asn202, Ile205, Ile206, Leu209, Leu214, Arg239, Asp298, Ala302, Thr306, Ile371, and Cys442 of CYP17A1. The major difference between TOK and TOKm was the bulk in the active site caused by substitution of leucine from alanine. This reduction in active site volume induced by Leu105 from alanine in TOKm did not alter the orientation of TOK-001 or any of its initial interaction with the CYP17A1 active site in TOKm complex. In this structure, the impact of the A105L mutation thus appears to be only steric in nature, as presented in abiraterone binding complex [15].

### 3.2 Stability of the AER, AERm, TOK, and TOKm during MD Simulation

**Fig. A.1:**
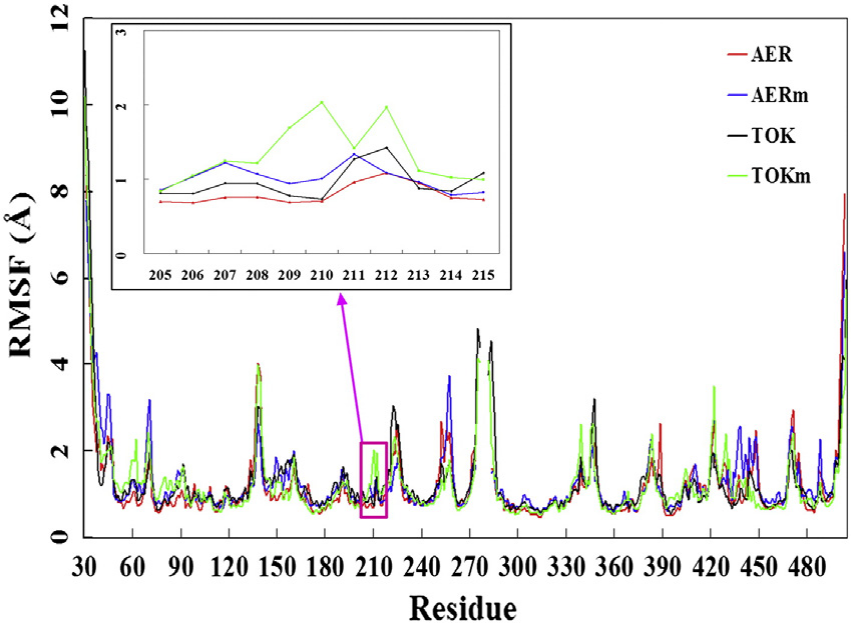
The calculated RMSF variations of Cα atoms of AER (red line), AERm (blue line), TOK (black line), and TOKm (green line).

**Fig. 3:**
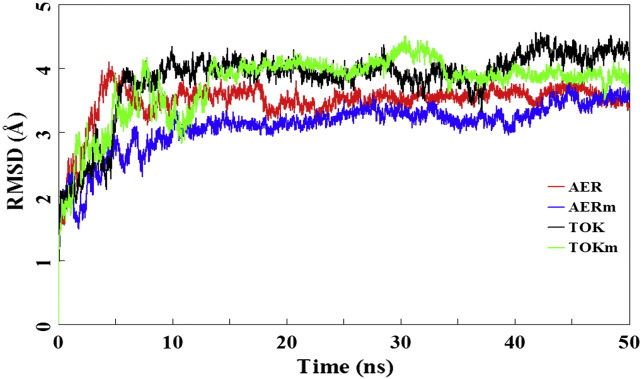
The RMSD values of C*a* atoms versus simulation time on four different complexes (AER, AERm, TOK, and TOKm).

During 50 ns of MD simulation, the root mean square deviation (RMSD) of all Cα atoms for four complexes (AER, AERm, TOK, and TOKm) were calculated to provide an overall measure of the departure of the structures from the initial coordinates as shown in Fig. 3. It is clear that the RMSD values are convergent and the systems remain in equilibrium during the 10–50 ns for AER, AERm, and TOK, while this value fluctuates until 13 ns for TOKm (see Fig. 3). With these regards, the results demonstrated that the global structures of four enzymes are relatively conserved during the last 37 ns of simulation. All subsequent conformational characteristic analysis of AER, AERm, TOK, and TOKm were performed on the last 37 ns of the simulation trajectories. Over the 50 ns trajectory, the RMSD for the abiraterone binding complexes continued to raise to a value around 3.55 Å (AER) and 3.27 Å (AERm) relative to the crystal structures, respectively, while those of the TOK-001 binding complexes were leveled around 4.00 Å. During the last 37 ns simulation, the Cα atoms RMSD values for all four enzymes stayed fairly low with the average RMSD values of 3.55 Å (standard deviation (SD): 0.11 Å), 3.27 Å (SD: 0.16 Å), 4.00 Å (SD: 0.20 Å), and 4.00 Å (SD: 0.15 Å) for AER, AERm, TOK, and TOKm, respectively. Most notably, the time evolution of the RMSD values in Fig. 3 indicates that the enzyme flexibility was significantly affected by A105L mutation of CYP17A1 in abiraterone binding systems, while it is not distinct in TOK-001 binding systems. To extend this analysis, the root mean square fluctuation (RMSF) values of Cα atoms calculation were also performed for MD simulation. The results suggest that the A105L mutation indeed induced some changes in RMSF variation in many regions as shown in Fig. A.1.

### 3.3 Multiple Comparisons among AER, AERm, TOK, and TOKm

Analysis of time-dependent atomic motion via MD simulation provides an effective means of exploring further inhibitor and enzyme interactions that are difficult to obtain from static structures alone, such as the crystal structures or the results of docking studies. In order to address structural differences at atomic-level between CYP17A1 and inhibitors i.e., abiraterone and TOK-001, and further investigate the effect of point mutation of CYP17A1 on the active site stability, and local interactions, namely, hydrophobic interaction, and hydrogen bonding across these four complexes (AER, AERm, TOK and TOKm) throughout MD simulation as described below.

### 3.4 Active Site Stability

**Fig. A.2:**
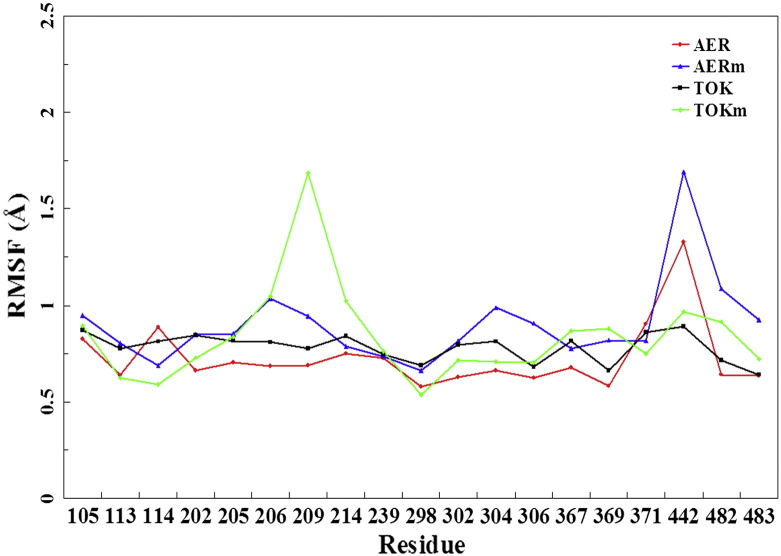
The calculated RMSF variations of Cα atoms of active site residues of AER (red line), AERm (blue line), TOK (black line), and TOKm (green line).

**Fig. A.3:**
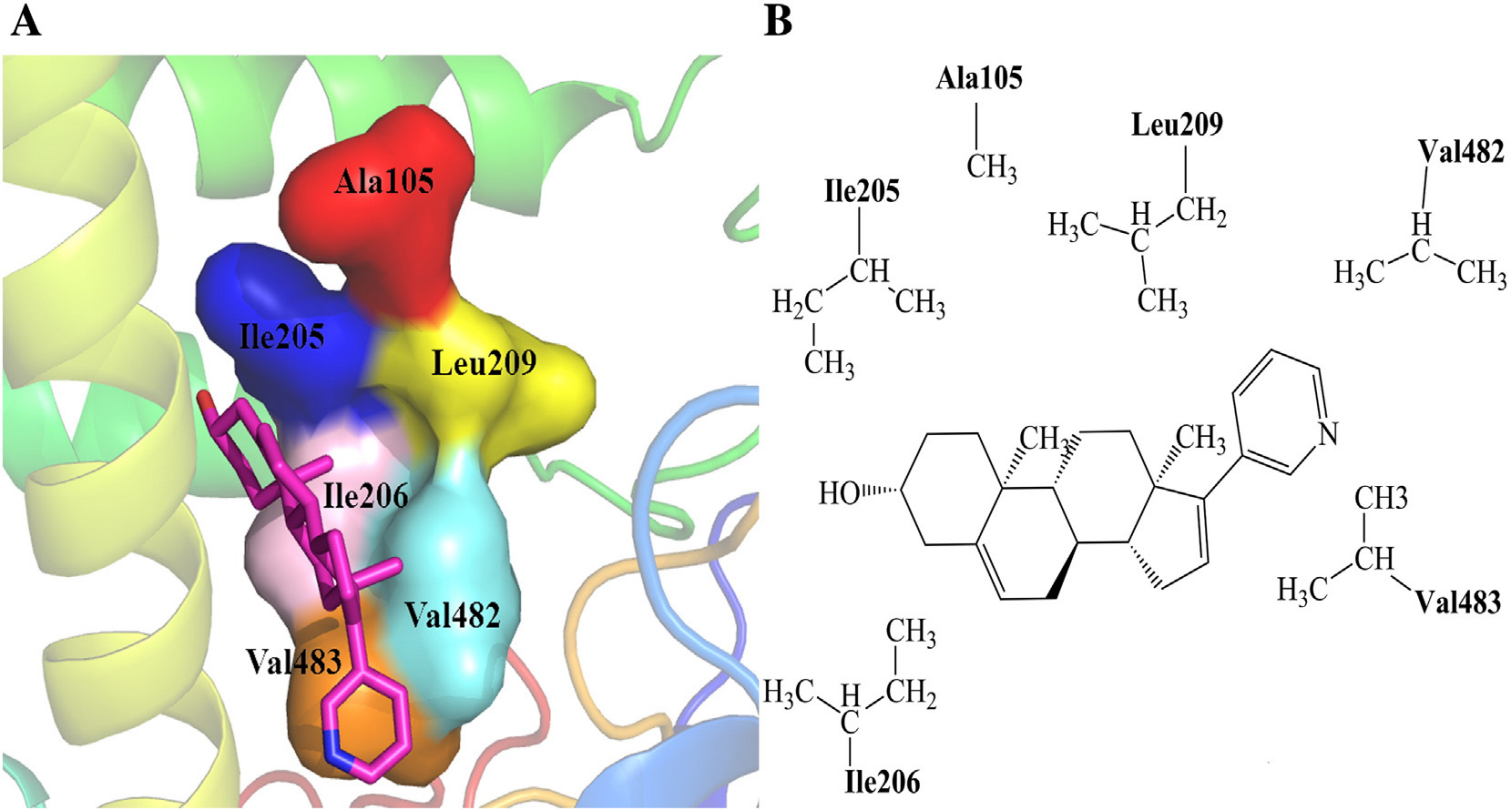
Illustration shows hydrophobic interaction network. (A) Residues involved in the hydrophobic interaction network are represented with colored surface while abiraterone is represented with magenta stick. (B) 2D representation of the hydrophobic interaction network is presented.

In order to characterize the effect of the A105L mutation upon conformational enzyme change of CYP17A1, here we used the RMSF analysis, which is capable of monitoring the local motion in the enzyme structure. Fig. A.2 shows the RMSF of Cα of binding site residues of the WT and MT CYP17A1 over 50 ns in a simulation run. As evidence from Fig. A.2, it is clear that A105L altered the binding site flexibility when compared to the WT, but the trends of mobility change of residues for those located in the active site are not entirely consistent. Residues for those located near Ala/Leu105, such as 205–214, 482, and 483, their mobility increases much according to the A105L mutation, while the other residues stay almost the same or decline when compared to the WT system. The reasonable explanation is a hydrophobic interaction network formed by residues 105, 205, 206, 209, 482, and 483 (Fig. A.3) in the WT system. The A105L mutation introduces interference to the existed network through the side chain of Leu105 involved in the network. While the other active site residues in AERm are not affected distinctly by this mutation, the effect caused by A105L mutation in TOK-001 binding system is more obvious than in abiraterone binding system as shown in Fig. A.2. The possible explanation is that abiraterone binds to the CYP17A1 more tightly than TOK-001, thus the residues for those interacted with abiraterone are more rigid than TOK-001.

#### 3.4.1 Local Interactions

**Fig. 4:**
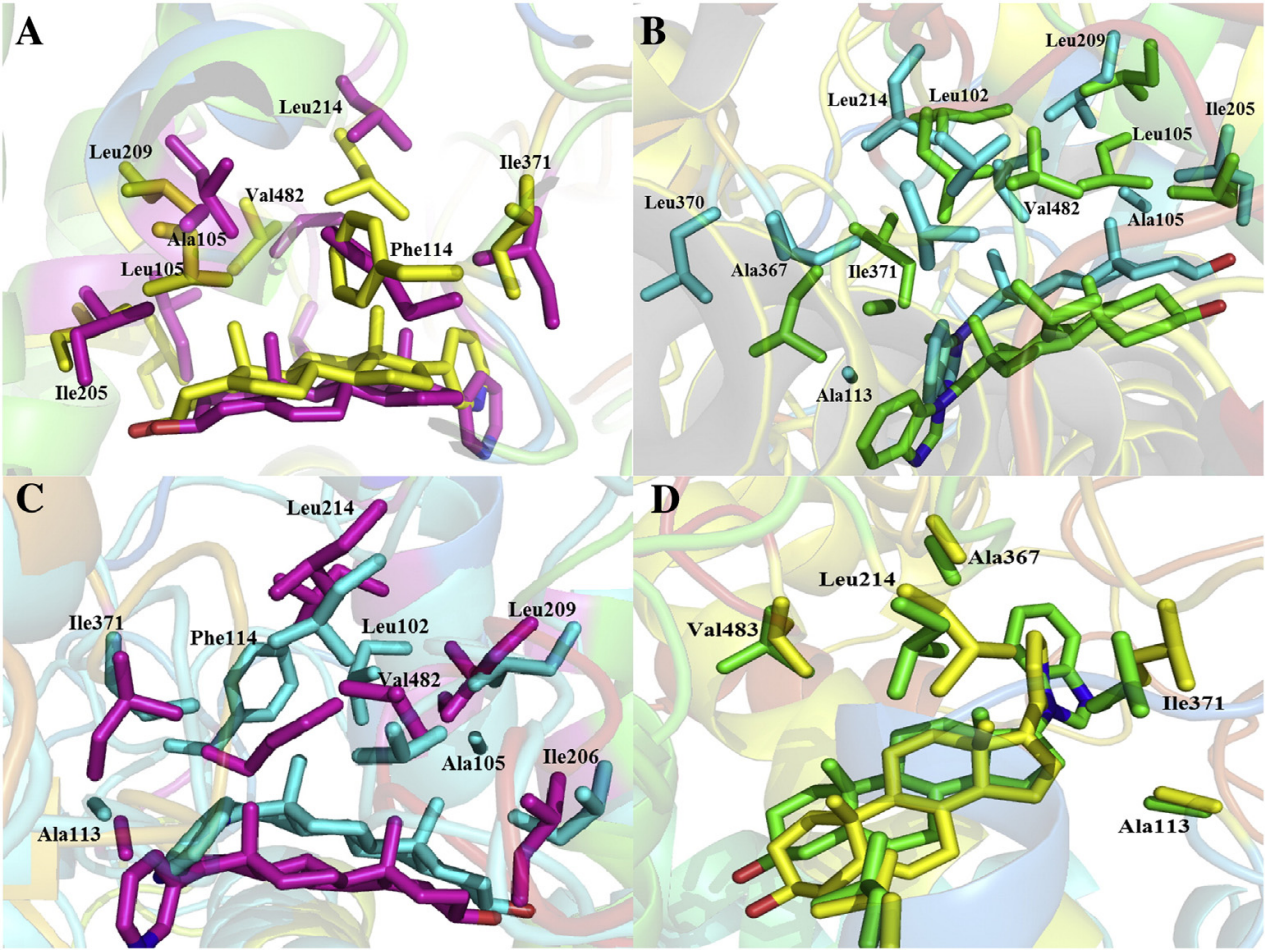
Illustration shows all pairwise complex structures comparisons of abiraterone and TOK-001 bound to WT and MT CYP17A1. (A) AER and AERm, (B) TOK and TOKm, (C) AER and TOK, (D) AERm and TOKm. CYP17A1 is represented with colored cartoon while inhibitors and active site residues from TOK (cyan), TOKm (green), AER (magenta), and AERm (yellow) are represented with colored stick.

Although the global structures were conserved between WT and MT CYP17A1, the local structural features are quite distinctive. The α-face positioned against the I-Helix, while the β-face of the steroidal ring, which included C18 and C19 methyl groups, interacts with hydrophobic residues around the binding site. Table 1 lists the crucial residues that made a large contribution to the inhibitor binding affinity for all four complexes. Several hydrophobic residues, such as Leu102, Ala/Leu105, Ala113, Phe114, Ala113, Ile206, Leu209, Leu214, Ala367, Ile371, Val482, and Val483, as well as the hydrophilic residue Asn202 were involved in the active pocket, and they also made a large contribution to the inhibitor binding. When the representative structures of these four complexes were superimposed with each other to obtain the preliminary estimation of the effect of mutation over the conformational change of the enzyme and inhibitor binding, this may suggest a molecular mechanism underlying the heterogeneities in conformational enzymes. The results are shown in Fig. 4.

#### 3.4.2 Hydrophobic Interaction

Hydrophobic is one of local interactions type. Since the active site of abiraterone and TOK-001 binding in CYP17A1 is mainly hydrophobic, the main contributions of inhibitors binding to CYP17A1 enzyme are the hydrophobic interactions formed between inhibitors and the active site non-polar residues. To investigate the differences between abiraterone/TOK-001 and CYP17A1, hydrophobic interactions of four complexes were analyzed in details. We first investigated the effect caused by A105L mutation to abiraterone binding. As shown in Fig. 4A and Table 1, when the representative structures of AER are compared with AERm, it is observed that the orientation of abiraterone remains the same in two complexes, whereas interactions and contributions between inhibitor and enzyme are distinct. The abiraterone forms stronger hydrophobic interactions with Phe114, Ile205 Ile206, Leu209, Ile371, and Val482 of WT CYP17A1 in AER, while most of those strong interactions reduced in AERm. The hydrophobic interactions between abiraterone and Leu105, Ala113, Leu214, Ala367, and Val483 of MT CYP17A1 mainly contributed into the inhibitor binding in AERm complex. In addition, the overall interactions contributing to inhibitor binding in AER were greater than AERm. For A105L mutation, in the presence of the bulk side chain, this residue extends its side chains to the space of existed hydrophobic interaction network as shown in Fig. A.3, leading to a series of successive movement of the hydrophobic residues that interact with abiraterone in MT CYP17A1, including Ala113, Ile206, and Leu209. Thus, the presence of the bulk side chain of Leu105 was not only to reduce the active site volume [15] but also to introduce the disruption of the hydrophobic interaction network. From the values of RMSD (see Fig. 3), we could see that the A105L mutation increased the global conformation stability of the enzyme; however it decreased the inhibitor and enzyme interactions.

By comparing of TOK and TOKm (Fig. 4B), we could see the hydrophobic interactions formed by TOK-001 and Leu102, Ala105, Phe114, Ile206, and Leu209 of WT CYP17A1, those mainly contributed into the inhibitor binding which were disappeared or weakened, whereas the interactions between TOK-001 and Ala113, Leu214, Ala367, Val482 and Val483 of MT CYP17A1 were strengthened in TOKm. The overall hydrophobic interactions contributing in TOKm were not altered according to the mutation. In addition to the variation of RMSD values of TOK and TOKm, we could conclude that the A105L mutation did not alter the binding of TOK-001 to CYP17A1 and the native conformation mobility of the enzyme distinctly.

To investigate the differences of these two inhibitors bound to WT CYP17A1, the comparisons of AER and TOK complexes are shown in Fig. 4C. In contrast to abiraterone, TOK-001 moved toward the hydrophobic pocket and formed a slightly larger volume between TOK-001 and I-helix, and the interactions between inhibitor and enzyme were distinct. In AER complex, Phe114, Ile206, Ile371, and Val482 of CYP17A1 formed hydrophobic interactions with abiraterone and made important contributions to abiraterone binding. Whereas, a distinct interaction network was detected in TOK, and Leu102, Ala105, Leu209, and Leu214 of CYP171 were involved. In addition, the RMSD value of AER of 3.55 (SD: 0.11 Å) was much lower than TOK of 4.00 Å (SD: 0.20 Å), and the overall hydrophobic interactions between the inhibitor and enzyme in AER were much stronger than TOK. These achieved results are consistent with the *in vitro* studies presented by Jacoby and Williams [42]. Their studies showed that TOK-001 activity could inhibit human CYP17A1, demonstrating the 17, 20-lyase (IC50 = 23 nmol l^− 1^) versus 17α-hydroxylase (IC50 = 73 nmol l^− 1^). In contrast, abiraterone activity showed more potent for CYP17A inhibition demonstrating 17, 20-lyase (IC50 = 12 nmol l^− 1^) and 17α-hydroxylase (IC50 = 7 nmol l^− 1^) [42].

In order to find out the differences between two inhibitors bound to MT CYP17A1, we superimposed the AERm and TOKm and made comparisons (Fig. 4D). In contrast to the large difference in binding between the two inhibitors bound to WT CYP17A1, abiraterone and TOK-001 showed similar overall interactions and contributions in MT complexes. The main contribution of hydrophobic interactions between abiraterone and Leu105, Leu214, and Ile371 of MT CYP17A1 in AERm almost equal to the interactions formed by TOK-001 and Ala113, Phe114, Ile205, Ile206, Ile209, Ala367, Val482, and Val483 of MT CYP17A1 in TOKm. In these two complexes, the inhibitors and binding site residues showed similar structural migrations.

#### 3.4.3 Hydrogen Bonding

**Fig. 5:**
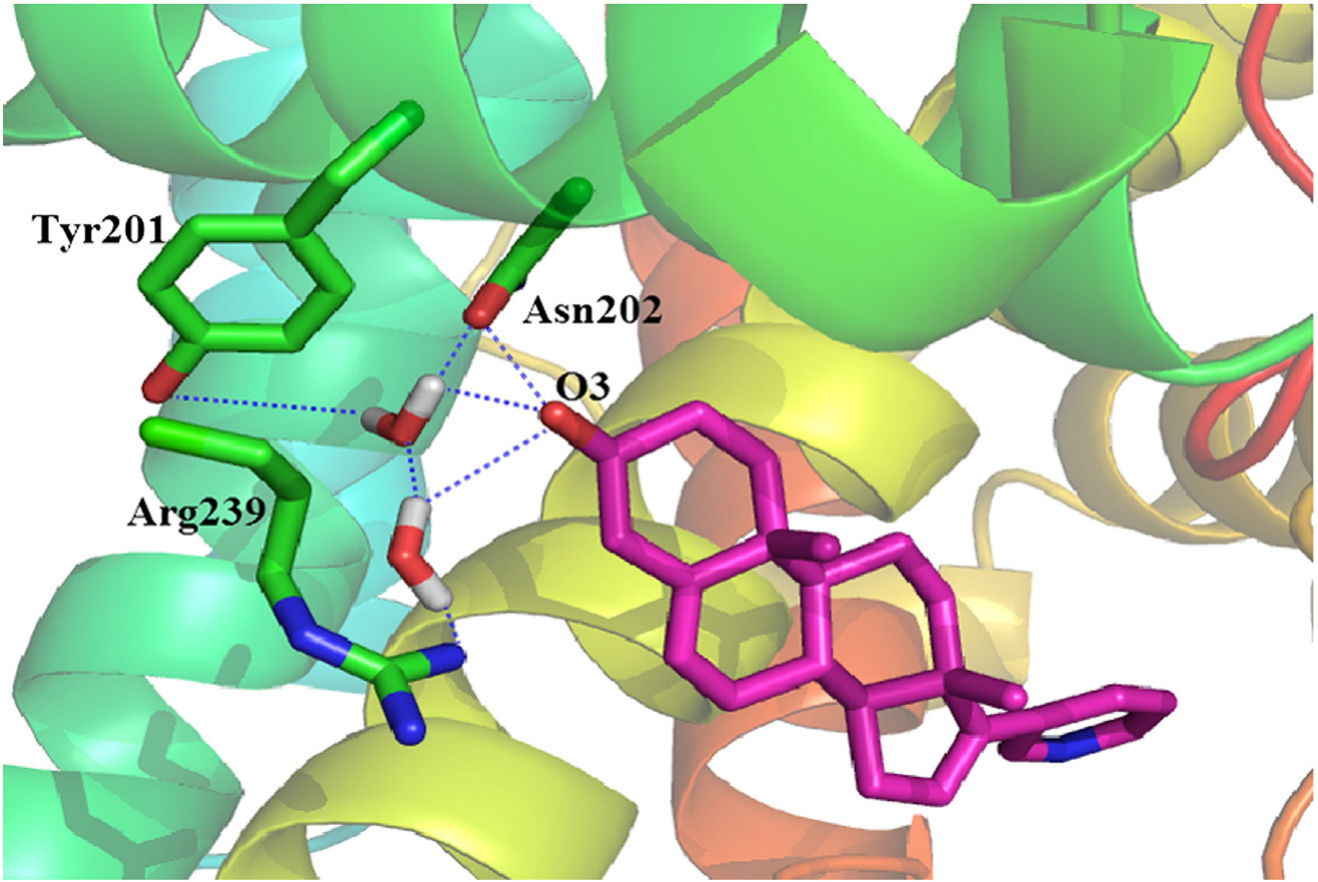
Illustration shows hydrogen bonding formed between CYP17A1 and inhibitor. Hydrogen bonding network of abiraterone bound to WT CYP17A1 at the top of the active site. Residues of CYP17A1, inhibitor, and water molecule that involved in the hydrogen bonding network are represented with colored stick. Blue dashed line represents hydrogen bonding.

Hydrogen bonding always plays an important role in inhibitor and enzyme binding. It is also the other type of local interactions. It would be ideal for the polar residues to have both hydrogen bonding and favorable packing interaction with the neighboring residues. Here, the single direct hydrogen bonding between inhibitor and the enzyme is part of a larger hydrogen bonding network. In all four complexes, this hydrogen bonding network involves Arg239, Tyr201, Asn202, inhibitors, and several conserved water molecules, as shown in Fig. 5. The only direct intermolecular hydrogen bonding, between the 3β-hydroxyl group of inhibitors and Asn202, is presented in all complexes except TOKm. The hydrogen bonding (acceptor H-donor atom distances of < 3.5 Å and acceptor . . . H-donor angles of < 60°) was reported when the occupancy was calculated of the percentage of time during simulation that the hydrogen bonding existed. Asn202 has important contributions to inhibitor binding through the formation of hydrogen bonding in approximately 70.06%, 18.16%, and 32.26% frames for AER, AERm, and TOK in the simulation trajectories, respectively, while this hydrogen bonding was not detected in TOKm. We could see that, both in abiraterone and TOK-001 binding complexes, this hydrogen bonding was weakened according to the A105L mutation. The most possible explanation is that the side chain of Leu105 introduces some disruption of the hydrophobic interaction network, and meanwhile weakened the hydrogen bonding. Further analysis on the representative structures of the inhibitor-CYP17A1 complexes suggests that hydrogen bonding may play an important role to stabilize the inhibitor binding. To investigate the strength of hydrogen bonding in four complexes during MD simulation, we further examined the hydrogen bonding distances between O3 of inhibitors and OD1 of Asn202 in the four complexes. This distance value remains relatively stable around 3.19 Å (SD: 0.40 Å) in AER; however, it fluctuates in a wider range in AERm for 4.40 Å (SD: 1.0 Å) and TOK for 4.15 Å (SD: 1.27 Å). For the simulation in TOKm, no hydrogen bonding was formed.

## 4 Conclusions

In conclusion, our study provides detailed atomistic insight into the structural differences of abiraterone and TOK-001 binding to WT and MT CYP17A1, as well as the effect on the inhibitors binding and conformational changes upon the A105L mutations of CYP17A1 for both abiraterone and TOK-001 binding. Considering multiple comparisons among four different complexes toward focusing on active site stability, interestingly in TOK complex, TOK-001 oriented toward the active pocket and formed larger volume with I-helix of CYP17A1 than abiraterone in AER complex. In contrast, AER complex showed tighter binding and more stability than TOK complex. For the local interactions between CYP17A1 and inhibitors, considering on hydrophobic interactions between abiraterone and residues of Phe114, Ile371,Val482 of CYP17A1 and the hydrogen bonding formed by O3 from abiraterone and OD1 from residue of Asn202 of CYP17A1 mainly contributed to the tighter binding than TOK-001.

This mainly resulted in the difference of these two complexes, nonetheless, the local interactions between abiraterone and CYP17A1 in AER complex weakened along with an increasing of the conformational mobility according to the A105L mutation. In comparison, the local interactions in TOK complex were even weaker than AER complex without affected distinctly by the A105L mutation on the conformational mobility. This suggests that the overall conformation of the CYP17A1 remains stable. This basic knowledge of inhibitor binding characteristics and key residues contributions can guide future effort on cytochrome P450 superfamily enzymes, so that desirable changes in their enzymatic activities may be achieved through changing an active site and/or an allosteric site of an enzyme. Additionally, this present study provides important insights into the effect of minor structural change on enzyme binding to different inhibitors, which can guide future experimental on efficient inhibitors design for CYP17A1, which provides theoretical basis of androgen-dependent disease therapy.

## Figures and Tables

**Table 1:** The percentages of four complexes across two local interaction types of hydrophobic interaction and hydrogen bonding during the MD simulation.

Interaction type	Atom	Occupied (%)				
Resid	AER	AERm	TOK	TOKm
Hydrophobic interaction	C18	102	0.00	0.00	25.72	0.00
113	0.14	8.22	2.18	17.38
114	43.18	5.42	15.48	9.12
214	0.04	72.68	22.28	40.66
367	0.14	27.16	0.12	44.14
371	52.82	25.46	10.62	4.62
482	63.40	4.60	17.14	9.90
483	0.22	2.72	0.00	12.68
C19	105	0.16	51.56	67.00	16.92
114	42.24	0.02	0.02	0.50
205	23.42	5.56	18.72	17.16
206	29.74	3.38	18.40	9.72
209	3.74	0.88	73.90	2.14
214	0.00	9.50	0.00	10.12
482	32.12	81.64	21.22	90.54
483	0.00	3.64	0.00	43.06
Hydrogen bonding	O3	202	70.06	18.16	32.26	0.00

Note: Resid stands for “Residue Id number.”
